# The impact of reward and punishment on skill learning depends on task demands

**DOI:** 10.1038/srep36056

**Published:** 2016-10-27

**Authors:** Adam Steel, Edward H. Silson, Charlotte J. Stagg, Chris I. Baker

**Affiliations:** 1 FMRIB Centre, Nuffield Department of Clinical Neurosciences, University of Oxford, Oxford, UK; 2 Laboratory of Brain and Cognition, National Institute of Mental Health, National Institutes of Health, Bethesda, MD, 20814, USA; 3 Oxford Centre for Human Brain Activity (OHBA), University Department of Psychiatry, University of Oxford, Oxford, UK

## Abstract

Reward and punishment motivate behavior, but it is unclear exactly how they impact skill performance and whether the effect varies across skills. The present study investigated the effect of reward and punishment in both a sequencing skill and a motor skill context. Participants trained on either a sequencing skill (serial reaction time task) or a motor skill (force-tracking task). Skill knowledge was tested immediately after training, and again 1 hour, 24–48 hours, and 30 days after training. We found a dissociation of the effects of reward and punishment on the tasks, primarily reflecting the impact of punishment. While punishment improved serial reaction time task performance, it impaired force-tracking task performance. In contrast to prior literature, neither reward nor punishment benefitted memory retention, arguing against the common assumption that reward ubiquitously benefits skill retention. Collectively, these results suggest that punishment impacts skilled behavior more than reward in a complex, task dependent fashion.

Reward and punishment, including biological reinforcers such as food, water, or pain, are important motivators for both human and animal behavior. The majority of neuroscience research has focused on studying the effects of reward and punishment on decision-making^1^,^2^,^3^. However, in recent years interest in using reward and punishment to augment motor skill learning has surged^4^,^5^,^6^,^7^,^8^,^9^ raising the enticing possibility that valenced feedback could be implemented in rehabilitation settings to improve physical therapy outcomes^10^,^11^,^12^,^13^. However, the variation in methodologies, performance metrics, and retention timescales used across different studies make establishing general principles challenging.

The present study examines the impact of reward and punishment on two different skill learning tasks: the serial reaction time task (SRTT), a sequencing task wherein participants press buttons in response to a stimulus appearing on a screen^14^; and the force tracking task (FTT)^15^,^16^,^17^, a motor task wherein participants squeeze a force transducer to follow a cursor on screen (Fig. 1A–D). The two tasks were implemented in as similar a manner as possible as possible to facilitate comparison between them. In an initial training session, participants trained on either the SRTT or FTT and received valenced feedback (monetary reward, monetary punishment, or motivated control [see methods]) based on their performance (calculated as [Mean Reaction Time/Accuracy per block] for SRTT; mean distance from the target per block in the FTT). In both tasks probe trials, during which stimuli were presented in either a fixed or a random order, were presented before and after training. General skill learning was assessed by comparing initial performance to performance after training, regardless of the probe block type. Sequence-specific skill learning was distinguished from general skill learning by comparing performance on fixed versus random probe blocks. Skill retention was then probed in the absence of feedback at 1 hour, 24 hours, and 30 days after completion of the training.

Participants were able to learn both tasks successfully and the skill learned was almost entirely retained at 30 days. Overall, we saw little effect of reward on either learning or retention. Punishment had no effect on skill retention, but had significant, task-dependent effects on learning. In the SRTT punishment improved speed with minimal impact on accuracy. In contrast, punishment impaired performance on the FTT. These results suggest that the effect of feedback varies depending on the skill being learned, and while feedback impacts online performance, the benefit of reward reported to retention may be less robust than previously demonstrated.

## Results

### Punishment improves online performance of the serial reaction time task

**Figure 1:**
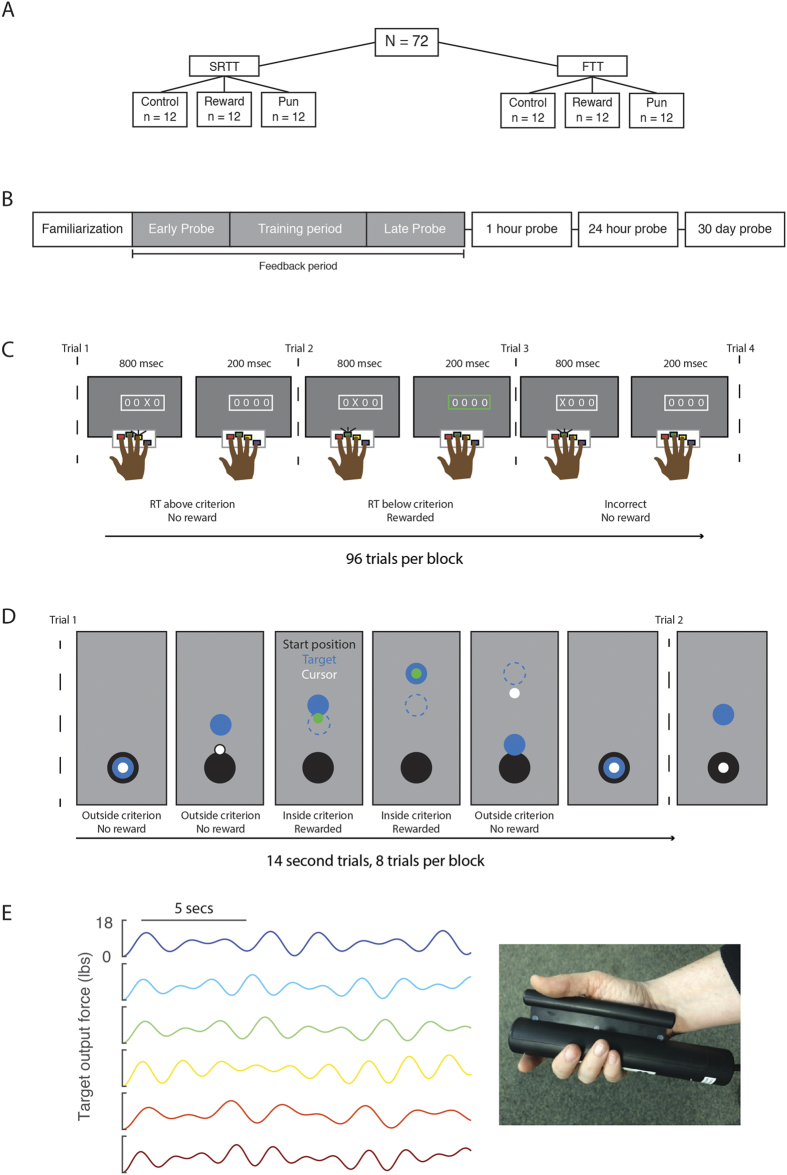
(**A**) Experimental design. Seventy-two participants were divided between two skill learning tasks: a task that demands integration of multiple memory systems, the serial reaction time task (SRTT), and a task that is learned primarily by the motor network, the force-tracking task (FTT). Within each task, participants were randomly assigned to three different feedback groups (reward, punishment, control). (**B**) Experimental timeline. For each task, trials were grouped into blocks of trials. Unbeknownst to the participants, during some blocks (“fixed sequence blocks”) the stimulus would appear according to a repeating pattern. During other periods the appearance of the stimulus was randomly determined (“random sequence blocks”). Following familiarization blocks, participants were trained on the task with valenced feedback. To assess sequence knowledge, training was bookended by early and late probes in which participants performed three blocks arranged random - sequence - random. Participants were then tested for sequence knowledge without feedback 1-hour, 24-hours, and 30-days after learning. (**C**) Serial reaction time task. Participants were presented with four locations on a screen denoted by “O’s”. A trial began when one “O” changed to an “X”. Participants were instructed to press the corresponding button on a controller as fast and accurately as possible. After 800 ms, the X changed back to an O, and participants were given valenced feedback for their performance on that trial. Performance in the SRTT was based on reaction time and accuracy of the button press. If participants were accurate and faster than they performed on their previous 96 trials, a participant would receive positive feedback (reward, or absence of punishment) on that trial. If they were slower or inaccurate, they would receive the negative outcome (either punishment or absence of reward). (**D**) Force-tracking task. Participants held a force transducer in their right hand and saw a black circle (start position), a blue circle (target), and a white circle (cursor). Participants were instructed to squeeze the force transducer to keep the cursor as close to the center of the target as possible. The target moved continuously during the trial (12 seconds), followed by a 2 second break between trials. The distance of the cursor from the target was the measure of performance. If the participant was closer to the center of the target than he were on their previous 8 trials, they would receive positive feedback. During sequence blocks the target followed one of six trajectories, (D, left) whereas during random blocks the target would follow a random trajectory.

**Figure 2:**
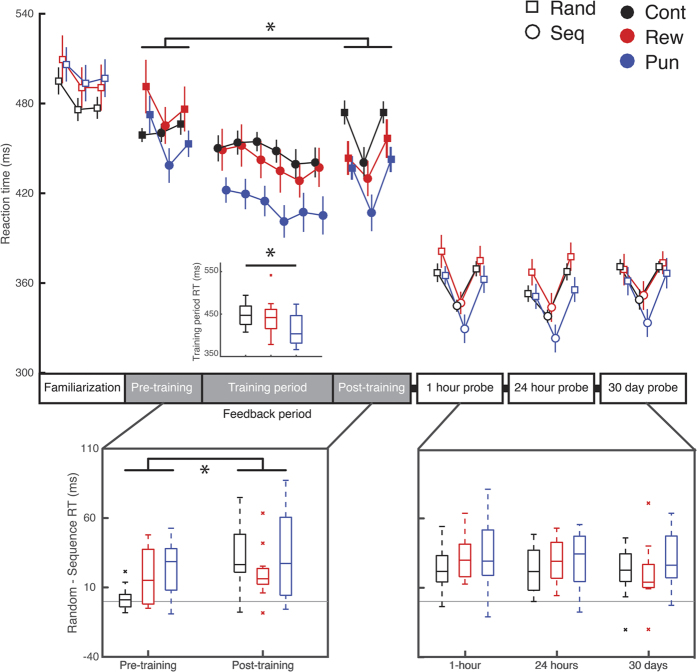
Punishment improves performance of the SRTT. During the training period, the punishment group was significantly faster than control overall (upper inset; Main effect Group: F_(2,33)_ = 3.286, p < 0.05; t_(22)_ = 2.884, p < 0.012). There was no difference between reward and control (t_(22)_ = 0.480, p = 0.636) or reward and punishment (t_(22)_ = 1.757, p = 0.093) during the training period. The reward group showed a greater reduction in average RT from pre- to post-training than control [Group x Time point interaction: F_(2,33)_ = 5.370, p < 0.01; Reward v Control: t_(22)_ = 3.730, p < 0.001; Punishment v Control: t_(22)_ = 2.199, p = 0.039, not significant considering multiple comparisons (α: 0.05/3 = 0.0167)]. There was also a significant Group x Timepoint x Sequence interaction (F_(2,33)_ = 5.370, p < 0.01). Both punishment and reward acquired more skill knowledge during the early sequence knowledge probe than control (F_(2,33)_ = 5.213, p < 0.05; punishment v control: t_(22)_ = 3.455, p < 0.005, reward v control: t_(22)_ = 2.545, p < 0.02), but did not differ from each other (reward v punishment: t_(22)_ = 0.707, p = 0.487). The reward group evidenced less sequence learning in the post-training probe than the pre-training probe (t_(22)_ = 2.884, p < 0.01), possibly due to the benefit of reward during the early learning period. The punishment group did not differ from control when correcting for multiple comparisons [t_(22)_ = 2.075, p = 0.05 (α: 0.05/3 = 0.0167)]. Feedback did not affect retention at any time point (lower right panel). Main panel shows mean ± SEM. Box plots show median, crosses show within group outliers. Asterisks denote periods with significant effects of feedback (p < 0.05).

We investigated the impact of reward and punishment on SRTT sequence learning in three different ways. First, we compared sequence knowledge during sequence knowledge probes either early in learning (immediately following familiarization when valenced feedback was first introduced) or late in learning (at the end of the training session) (see Fig. 1). During these probes, we estimated sequence knowledge by calculating the reaction time (RT) difference between fixed and random blocks (Fig. 2). A repeated measures ANOVA, with Group (reward, punishment, control), Sequence (fixed, random) and Time-point (early, late) as factors revealed a significant three-way interaction between Group, Sequence, and Time-point (F_(2,33)_ = 5.370, p < 0.01). Follow-up analyses indicated that both punishment and reward groups acquired more skill knowledge during the early sequence knowledge probe than control (F_(2,33)_ = 5.213, p < 0.05; punishment v control: t_(22)_ = 3.455, p < 0.005, reward v control: t_(22)_ = 2.545, p < 0.02), but did not differ from each other (reward v punishment: t_(22)_ = 0.707, p = 0.487). Further, the control group evidenced a greater gain in sequence knowledge from the early- to late sequence knowledge probe compared to reward (t_(22)_ = 2.884, p < 0.01), although this comparison was not significant for control versus punishment when correcting for multiple comparisons (t_(22)_ = 2.075, p = 0.05), in part reflecting the benefit of feedback to early learning. These results suggest that feedback facilitates rapid sequence learning on the SRTT.

Second, to examine the effect of valenced feedback on learning rate, we compared the median reaction time across the six consecutive sequence training blocks immediately following the early sequence knowledge probe using a repeated measures ANOVA with Block (1–6) and Group as factors. Participants showed improvement over the course of training (Main effect of Block: F_(5,33)_ = 11.224, p < 0.001). We also found a main effect of Group (F_(2,33)_ = 3.286, p < 0.05) and follow up tests indicated that the punishment group was significantly faster than control overall (punishment versus control: t_(22)_ = 2.884, p < 0.012), but there was no difference between reward and control (t_(22)_ = 0.480, p = 0.636), or reward and punishment (t_(22)_ = 1.757, p = 0.093, two-tailed) during the training period. The lack of a significant Group by Sequence interaction in the post- probe highlights that this is a general, rather than sequence-specific, improvement.

Finally, we examined the impact of valenced feedback on retention. All groups demonstrated retention of sequence knowledge at all time-points (Main effect Sequence: F_(1,33)_ = 100.245, p < 0.001; t_(35)_ = 10.036, p < 0.001). There was no influence of feedback Group on retention.

Collectively, these results show that both reward and punishment increased early learning of the sequence with punishment additionally having a marked effect on performance during training.

### Punishment impaired performance of the force-tracking task

**Figure 3:**
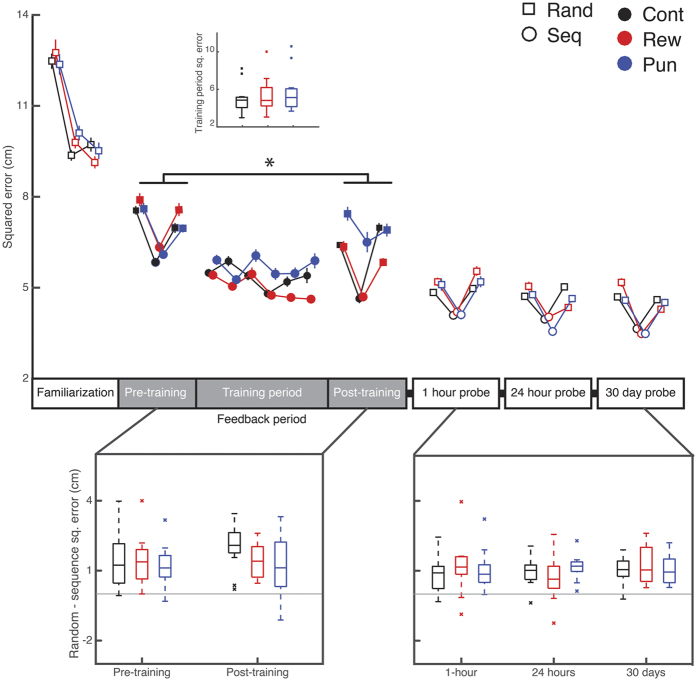
Punishment impairs to performance on the FTT. Compared to reward, punishment impaired general performance improvement from the pre- to post- training probe (Probe x Group interaction (F_(2,33)_ = 3.526, p < 0.05; t_(35)_ = 2.372, p < 0.03). Reward was not beneficial compared to control (t_(35)_ = 1.688, p = 0.106. Punishment did not impair performance compared to control (t_(35)_ = 1.206, p = 0.241). Unlike the SRTT, feedback had no influence on sequence specific knowledge (lower left) or performance during the training period (inset). As was found in the SRTT, feedback did not affect performance during the retention probes (lower right). Main panel shows mean ± SEM. Box plots show median, crosses show within group outliers. Asterisks denote periods with significant effects of feedback (p < 0.05). One outlier from punishment in the post-training period is not pictured in the boxplot [Random – Sequence sq err. = −7 cm sq err].

We conducted the same three analyses on data from FTT (Fig. 3A,B). A description of the trial-by-trial performance in the FTT is available in the supplemental materials. First, sequence knowledge during the feedback period was evaluated by comparing the mean squared error during sequence and random blocks in the early and late sequence knowledge probes using repeated measures ANOVA (Time-point x Sequence x Group). There was a significant interaction between Time-point and Group (F_(2,33)_ = 3.526, p < 0.05), but in contrast to the SRTT, no three-way interaction with Sequence (F_(2,33)_ = 1.212, p = 0.310). Follow-up analyses indicated that only reward improved from pre- to post- training time-point (pre- versus post- probe, 1-sample t-test, reward: t_(11)_ = 4.250, p < 0.001, punishment: t_(11)_ = 0.100, p = 0.922, control: t_(11)_ = 2.292, p = 0.043 [n.s. corrected for multiple comparisons]). Punishment showed significantly less improvement than the reward group from the pre- to post- training probe (t_(35)_ = 2.372, p < 0.03) but was not significantly worse than control (t_(35)_ = 1.206, p = 0.241). There was no difference between reward and control (t_(35)_ = 1.688, p = 0.106). Unlike the SRTT, there was no significant Time-Point x Sequence x Group interaction, suggesting that feedback modulated general, rather than sequence-specific, learning on the FTT.

Second, we examined performance during the six consecutive sequence training blocks using repeated measures ANOVA, with Block and Group as factors. All feedback groups showed improvement across the training period (Main effect of Block: F_(5,165)_ = 8.478, p < 0.001; S2 versus S7: t_(35)_ = 2.836, p < 0.01). Although reward tended to outperform punishment during training, there was no effect of Group on learning rate in the FTT (Group x Block: F_(10,165)_ = 1.186, p = 0.156).

Finally, we examined the effect of valenced feedback on retention in the FTT. Five participants did not complete the retention probes due to timetabling. This left us with 10 control, 9 reward, and 11 punishment participants for retention analyses. All groups demonstrated retention of sequence knowledge at all time-points (Main effect of Sequence, F (1, 27) = 86.387, p < 0.001; t_(35)_ = 9.030, p < 0.001). There was no main effect or interaction with feedback Group on retention.

Collectively, these results show that the primary effect of feedback in FTT was for punishment to impair learning from the pre- to post-training probe time points.

## Discussion

This study sought to determine whether the impact of reward and punishment generalizes across different types of motor skill learning, as implemented using a Serial Reaction Time Task (SRTT) and a Force Tracking Task (FTT). We found that punishment had opposing effects on performance of the two skills. During performance of the SRTT, training with punishment led to improved reaction times overall with minimal detriment to accuracy. In contrast, punishment impaired performance of the FTT. These effects were only present whilst feedback was being given; there was no effect of training with feedback on general or sequence-specific retention measured at 1 hour, 24 hours, and 30 days in either task. Our results refute any simple model of the interaction between feedback and performance. Instead, we show that the impact of feedback depends on the training environment and the skill being learned.

There may be a number of reasons for this task-specific effect of feedback. While both tasks rely on sequence learning, they differ with respect to the mechanism that facilitates improvement. The motivational salience of punishment (i.e. loss aversion) may explain the performance benefit seen on the SRTT, where the added attention facilitated by punishment has been hypothesized to recruit additional neural resources to aid SRTT performance^8^,^18^. However, a purely motivational account cannot explain the deleterious effect of punishment to performance on the FTT. Therefore, we need to consider alternative explanations that may account for the differential effects of reward and punishment to performance these two tasks.

The two tasks also differ with respect to their motor demands. Specifically, in our implementation, performance on the FTT relies on more precise motor control than the SRTT. Within the motor system, others have reported that reward-related dopaminergic activity reduces motor noise^19^, while dopaminergic activity associated with punishment leads to an increase in motor variability, i.e. noise^20^. We found that punishment impaired general (i.e. non sequence-specific) performance on the FTT. After one-hour, during the retention test without feedback, the punishment group performed as well as the reward and control groups. We think that our findings are consistent with the hypothesis that punishment may increase motor noise, which may have led to impaired performance by the punishment group during training. Because increased motor variability was not directly measured in our implementation of the SRTT, participants would not be penalized for any variation in movement that did not impact reaction time directly. If an assessment of motor variability was considered in the evaluation of SRTT performance, one might find that punishment impairs this dimension of performance. Our implementation of the SRTT and the FTT do not have a direct measure of motor variability and we cannot explicitly address this issue in the present study. Future work should examine this question.

The implementations of the tasks used here also differed with respect to the information content of a given instance of feedback. Ordinarily, learning on the SRTT relies on the positive prediction error encoded in the striatum that occurs on fixed-sequence trials^8^,^21^. The reward or punishment in the SRTT may augment this positive prediction error and facilitate performance and learning. In contrast, the moment-to-moment feedback given on the FTT is not associated with an instantaneous positive prediction error signal. Rather, our implementation of the FTT is similar to discontinuous motor tasks that rely on the cerebellum and may therefore not benefit from moment-to-moment feedback^22^ (but also see Galea, *et al.*^4^ for an additional account of cerebellar learning with feedback). Finally, although information content was not intentionally manipulated, this difference may also alter effect the reward and punishment on these tasks.

Unlike prior studies, we saw no benefit of reward to retention^4^,^7^,^8^,^10^. Most studies that have looked at reward and punishment in skill learning have only examined immediate recall^4^,^8^,^10^, and only one study has shown a benefit of reward to long-term retention of a motor skill^7^. In their study, Abe, *et al.*^7^ observed that the control and punishment groups evidenced diminished performance after 30-days compared to their post-training time-point. Importantly, Abe, *et al.*^7^ also found that the reward group showed offline gains from the immediate time point to 24-hours after training, and this effect persisted through 30-days. So, while in our study the punishment and control group did not evidence forgetting from 24-hours to 30-days, potentially limiting our sensitivity to the effect of reward, the reward group in our study also did not show any offline-gains. As such, we are confident in our finding that reward did not impact retention.

While not discussed at length by Abe and colleagues, their punishment group performed significantly worse during training, suggesting that the skill was not learned as effectively by participants in that group. Therefore, it is unclear whether the difference in memory observed in their study can be attributed to a benefit of reward to consolidation or to ineffective acquisition when training with punishment. Our study design differed from the implementation used by Abe and colleagues^7^ with respect to the input device (whole-hand grip force in our study, precision pinch force by Abe and colleagues), feedback timing, and trial duration. However, our result questions robustness of the finding that reward benefits skill retention. We maximized our design to be sensitive to differences in online-learning rather than retention, and future studies should examine other factors that influence the effect of feedback on retention of skill memories.

With respect to the SRTT, it is worth considering that our participants evidenced less sequence-specific learning than some others have found in unrewarded versions of this task, where the difference between sequence and random trials can be up to 80 ms^23^,^24^,^25^. However, there is considerable variability in the difference between sequence and random trials on the SRTT reported in the literature, and some groups have reported sequence-specific learning effects on the SRTT to be between 10 and 30 ms^26^,^27^. The difference reported after learning by the Control, Reward, and Punishment groups in our study is approximately equal to the difference for the rewarded group reported by Wachter, *et al.*^8^ (~30 ms) and more than observed in their control and punishment groups. This is evidence of substantially less sequence-specific knowledge than we observed in our study, and we are therefore confident that participants were able to learn and express sequence-specific knowledge in all three feedback conditions.

Finally, we recognize that there are difficulties in comparing performance across tasks. Because the tasks used here vary in performance outcome (response time in the SRTT, tracking error in the FTT), comparing them in a quantitative way is not possible. However, the dissociation in the effect of punishment in these contexts provides compelling evidence that the effect does depend on task. Moreover, our study brings together the previously disparate literature examining the effects of reward and punishment on skill learning. This result shines light on the challenge of extrapolating from a single experiment in a specific context to a more general account of skill learning.

Overall, we have demonstrated that punishment modulates on-line performance in a task-specific manner and in our study we found that neither reward nor punishment modulates long-term retention of skill memories. These findings cast doubt on the commonly held hypothesis that reward is ubiquitously beneficial to memory, and, suggest that the interaction between feedback and learning should be better understood before feedback can be fully exploited in clinical situations.

## Materials and Methods

The study design was the same for both tasks (Fig. 1A). Participants trained on either the serial reaction time task (SRTT), or the force-tracking task (FTT). For both tasks, trials were presented over 15 blocks. A 30-second break (minimum) separated each block of trials. Unbeknownst to the participants, during some blocks (“fixed sequence blocks”) the stimulus would appear according to a repeating pattern (described below for each task). During other periods the appearance of the stimulus was randomly determined (“random sequence blocks”).

Familiarization and training blocks were conducted in the bore of an MRI scanner. To acclimatize participants to the task, and establish their baseline level of performance, the task began with three random-sequence blocks without feedback (“familiarization blocks”). Participants were unaware of the forthcoming feedback manipulation during these familiarization blocks. Then the feedback period began, starting with a pre-training probe (three blocks, random – fixed – random), then the training blocks (six consecutive fixed-sequence blocks), and, finally, a post-training probe (three blocks, random – fixed – random). The difference in performance between the average of the two random blocks, versus the fixed sequence block, during the probes was used to index sequence knowledge^28^.

To test the impact of reward and punishment on skill learning, participants were randomised into one of 3 feedback groups: reward, punishment, or uninformative (control). During the feedback period, reward, punishment, or control feedback was provided based on the participant’s ongoing performance. The feedback paradigm for each task is outlined separately below.

Participants were given retention probes at one-hour, 24–48 hours, and 30 days after training. No feedback was delivered during the retention probes. The second probe always occurred after at least one night’s sleep.

The initial visit (Familiarization, Early probe, Learning, and Late Probe) took place while participants underwent MRI scanning.

### Participants

78 participants (47 female, mean age = 25 years ± 4.25 std.) participated in this experiment. All participants were right-handed, free from neurological disorders, and had normal or corrected-to-normal vision. All participants gave informed consent and the study was performed with National Institutes of Health Institutional Review Board approval in accordance with the Declaration of Helsinki (93-M-0170, NCT00001360). Data from six individuals were removed from the study due to inattention (defined as non-responsive or inaccurate on greater than 50% of trials during training) or inability to complete the training session.

### Serial reaction time task (SRTT)

The version of the SRTT used here adds feedback to the traditional implementation. At the beginning of each block participants were presented with four “O”s, arranged in a line, at the centre of the screen. These stimuli were presented in white on a grey background (Fig. 1B). A trial began when one of the “O”s changed to an “X”. Participants were instructed to respond as quickly and accurately as possible, using the corresponding button, on a four-button response device held in their right hand. The “X” remained on screen for 800 ms regardless of whether the participant made a response, followed by a 200 ms fixed inter-trial interval, during which time the four “O”s were displayed.

A block consisted of 96 trials. During fixed-sequence blocks, the stimuli appeared according to a fixed 12-item sequence repeated 8 times (e.g. 3–4–1–2–3–1–4–3–2–4–2–1). Each fixed block began at a unique position within the sequence, to help prevent explicit knowledge of the sequence from developing^29^. In the random blocks, the stimuli appeared according to a randomly generated sequence, without repeats on back-to-back trials, so, for example, participants would never see the triplet 1–1–2.

Between each block, participants saw the phrase “Nice job, take a breather”. After five seconds, a black fixation-cross appeared on the screen for 25 seconds. Five seconds before the next block began, the cross turned blue to cue the participants that the block was about to start.

During the retention probes, participants performed three blocks (random – fixed – random on a 15-inch Macbook Pro using a button box identical to the one used during training. During these retention probes, the next trial began 200 ms after the participant initiated their response. No feedback was given during the retention blocks. The first button press made after stimulus presentation was considered the participant’s response. All responses were included in the analysis. Any missed trial was counted as an error, and only correct trials were considered for analysis of RTs.

### Force-tracking task

In the force-tracking task (FTT), participants continuously modulated their grip force to match a target force output^16^,^17^. In the traditional implementation, participants are exposed to a single pattern of force modulation repeated each trial. This design does not allow discrimination between general improvement (i.e. familiarization with the task and/or the force transducer) and improvement specific to the trained sequence of force modulation. Therefore, we decided to adapt the traditional FTT method to align it with the experimental design that is traditional for the SRTT, i.e. by including random sequence blocks.

A given trial consisted of a 14 second continuous pattern of grip modulation. At the beginning of a trial, participants were presented with three circles on a grey background projected onto a screen: a white circle (Cursor, 0.5 cm diameter), a blue circle (Target, 1.5 cm diameter), and a black circle (bottom of the screen, 2 cm diameter, indicating the position corresponding to minimum pressure; Fig. 1C). Participants held the force transducer (Current Designs, Inc., Philadelphia, PA) in the right hand between the four fingers and palm (Fig. 1D, right). Participants were instructed to squeeze the force transducer (increasing force moving the cursor upwards) to keep the cursor as close to the center of the target as possible as the target moved vertically on the screen. During fixed blocks, participants were randomly assigned to one of six sequences (Fig. 1D, left). During random blocks, the target followed a trajectory generated by the linear combination of four waveforms, with periods between 0.01 and 3 Hz. These waveforms were constrained to have identical average amplitude (target height), and the number and value of local maxima and minima were constant across the random blocks.

For data analysis, the squared distance from the cursor to the target was calculated at each frame refresh (60 Hz). The first 10 frames were removed from each trial. The mean of the remaining time points was calculated to determine performance, and trials were averaged across blocks.

### Feedback

All participants were paid a base remuneration of $80 for participating in the study. At the start of the feedback period, participants were informed they could earn more money based on their performance.

During the feedback period, participants were given either reward, punishment, or control feedback. The presence of reward or the absence of punishment was based on participant’s performance. In both the SRTT and the FTT, an initial criterion was defined, based on the participant’s median performance during the final familiarization block. As participants progressed through training, this criterion was re-evaluated after each block, to encourage continuous improvement. In the reward group, the feedback indicated that the participant’s performance was getting better at the task. In the punishment group, the feedback indicated they were getting worse. Because the frequency of feedback events differed between the reward and punishment groups (reward from high-to-low as training progressed, punishment from low-to-high), the control group was split into two different sub-groups (control-reward and control-punishment). The control groups received feedback at a frequency that matched the corresponding feedback group but was not related to their performance. Participants in the control group were made aware that the feedback was not meaningful. We considered the reward and punishment control groups together in the analyses, as is typical in these studies^7^,^8^.

In the SRTT, performance was defined as the accuracy (correct or incorrect) and reaction time (RT) of a given trial. Feedback was given on a trial-by-trial basis (Fig. 1C). This was indicated to the participant when the white frame around the stimulus changed to green (reward) or red (punishment). In the reward group, the participants were given feedback if their response was accurate and their RT was faster than their criterion RT, which indicated that they earned money ($0.05 from a starting point of $0) on that trial. In the punishment group, participants were given feedback if they were incorrect, or their RT was slower than their criterion, which indicated that they lost money ($0.05 deducted from a starting point of $55) on that trial. Participants in the control-reward and control-punishment groups saw red or green colour changes, respectively, at a frequency matched to punishment and reward, respectively. Control participants were told that they would be paid based on their speed and accuracy. Importantly, to control for the motivational differences between gain and loss, participants were not told the precise value of a given trial. This allowed us to assess the hedonic value of the feedback, rather than the level on a perceived-value function. Between blocks, for the reward and punishment groups, the current earning total was displayed (e.g. “You have earned $5.00”). Control participants saw the phrase, “You have earned money”. The criterion RT was calculated as median performance in the first familiarization block. After each block, the median + standard deviation of performance was calculated, and compared with the criterion. If this test criterion was faster (SRTT) or more accurate (FTT) than the previous criterion, the criterion was updated. During the SRTT, only the correct responses were considered when establishing the criterion reaction time.

Feedback in the FTT was based on the distance of the cursor from the target (Fig. 1C). For the reward group, participants began with $0. As participants performed the task, their cursor turned from white to green when the distance from the target was less than their criterion. This indicated that they were gaining money at that time. In the punishment group, participants began with $45, and the cursor turned red if it was outside their criterion distance. This indicated that they were losing money. For reward-control and punishment control, the cursor changed to green or red, respectively, but was unrelated to their performance. For control, the duration of each feedback instance, as well as cumulative feedback given on each trial, was matched to the appropriate group. Between each block, participants were shown their cumulative earnings. Control participants saw the phrase “You have money”.

### Statistical analyses

In both tasks, the six training blocks were compared using a repeated-measures ANOVA to establish differences in learning rate (Block x Group). Learning was indexed by comparing the performance (RT and accuracy separately for SRTT; squared distance from the target [squared error] for FTT) on the sequence blocks to the average of the two random blocks at the pre and post training time points using a repeated-measures ANOVA (Time point x Sequence x Group). Memory for the sequence was evaluated by comparing the fixed block, to the mean of the two random blocks, at each retention time point using a repeated-measures ANOVA (Time point x Sequence x Group). A Bonferroni correction was applied for post-hoc analyses to correct for multiple comparisons. If sphericity was violated, the Hyunh-Feldt correction was applied.

## Additional Information

**How to cite this article**: Steel, A. *et al.* The impact of reward and punishment on skill learning depends on task demands. *Sci. Rep.* **6**, 36056; doi: 10.1038/srep36056 (2016).

**Publisher’s note:** Springer Nature remains neutral with regard to jurisdictional claims in published maps and institutional affiliations.

## Supplementary Material

Supplementary Information
