# Supplementary material for: The impact of reward and punishment on skill learning depends on task demands
Source: Sci Rep. 2016 Oct 27;6:36056. doi: 10.1038/srep36056 (PMC5081526; doi:10.1038/srep36056)
Supplement: Supplementary Information [file srep36056-s1.pdf]

The impact of reward and punishment on skill learning depends on task demands

Supplemental Materials

Adam Steel[1,2], Edward Silson[2], Charlotte J. Stagg[1,3$], Chris I. Baker[2,$]

Supplemental materials

S1. Earning and feedback data

The amount of money earned in the reward and punishment groups did not differ in the SRTT ($t_{(22)} = 0.096$, $p = 0.924$), but did differ in number of feedback instances ($t_{(22)} = -5.604$, $p < 0.001$), as punishment was significantly better at avoiding negative feedback. Reward and punishment did not differ significantly in the amount of feedback received during FTT ($t_{(22)} = 0.275$, $p = 0.786$), but did differ in total amount earned (Reward = \$19.55 ± 0.45, Punishment = \$25.75 ± 1.08; $t_{(22)} = -5.684$, $p < 0.001$).

S2. General learning measures

SRTT

We first wanted to ensure that the subjects were able to learn the task. The results for the RT data are presented in Figure 2 A and B. To investigate the level of sequence-specific knowledge in the SRTT, we compared the difference in RT between fixed and random blocks during the pre- and post- training probes using a repeated measures ANOVA, with Group (reward, punishment, control), Sequence (fixed, random) and Time point (pre, post) as factors. As expected, participants were faster in both the fixed and random sequence blocks post-, compared to pre-, training (Main effect of Time point: $F_{(1,33)} = 30.903$, $p < 0.001$). We then went on to explore sequence-specific knowledge and, as expected, participants were faster during fixed than random sequence blocks (Main effect Sequence: $F_{(1,33)} = 58.412$, $p < 0.001$). Furthermore, sequence-specific knowledge increased over time (Time point x Sequence interaction: $F_{(2,33)} = 9.291$, $p < 0.005$). To explore this interaction further, we compared the differences between sequence and random in the pre- and post-

training probes and found that this difference was greater in the post training probe than the pre training probe ($t_{(35)} = 2.815$, $p < 0.01$).

FTT

Unlike the traditional implementation of the FTT, our design allowed us to differentiate between sequence knowledge and general task improvement. Sequence knowledge during the feedback period in the FTT was evaluated by comparing the mean squared error during sequence and random blocks during the early and late probes using a repeated measures ANOVA (Time point x Sequence x Group). As in the SRTT, subjects showed a general improvement from early to late probe (Main effect of Time point: $F_{(1,33)} = 8.222$, $p < 0.01$; $t_{(35)} = 2.782$, $p < 0.01$) , suggesting a non sequence-specific improvement, and were more proficient at the fixed compared to the random blocks (Main effect of Sequence: $F_{(1,33)} = 54.829$, $p < 0.001$; $t_{(35)} = 7.267$, $p < 0.001$) during both probes . There was no significant sequence by time point interaction (Main effect of Time point: $F_{(1,33)} = 0.013$, $p = 0.908$), suggesting that the improvement seen was in general performance, rather than being sequence-specific.

S3. *Reward and punishment did not differ in error rate during training in the SRTT.*

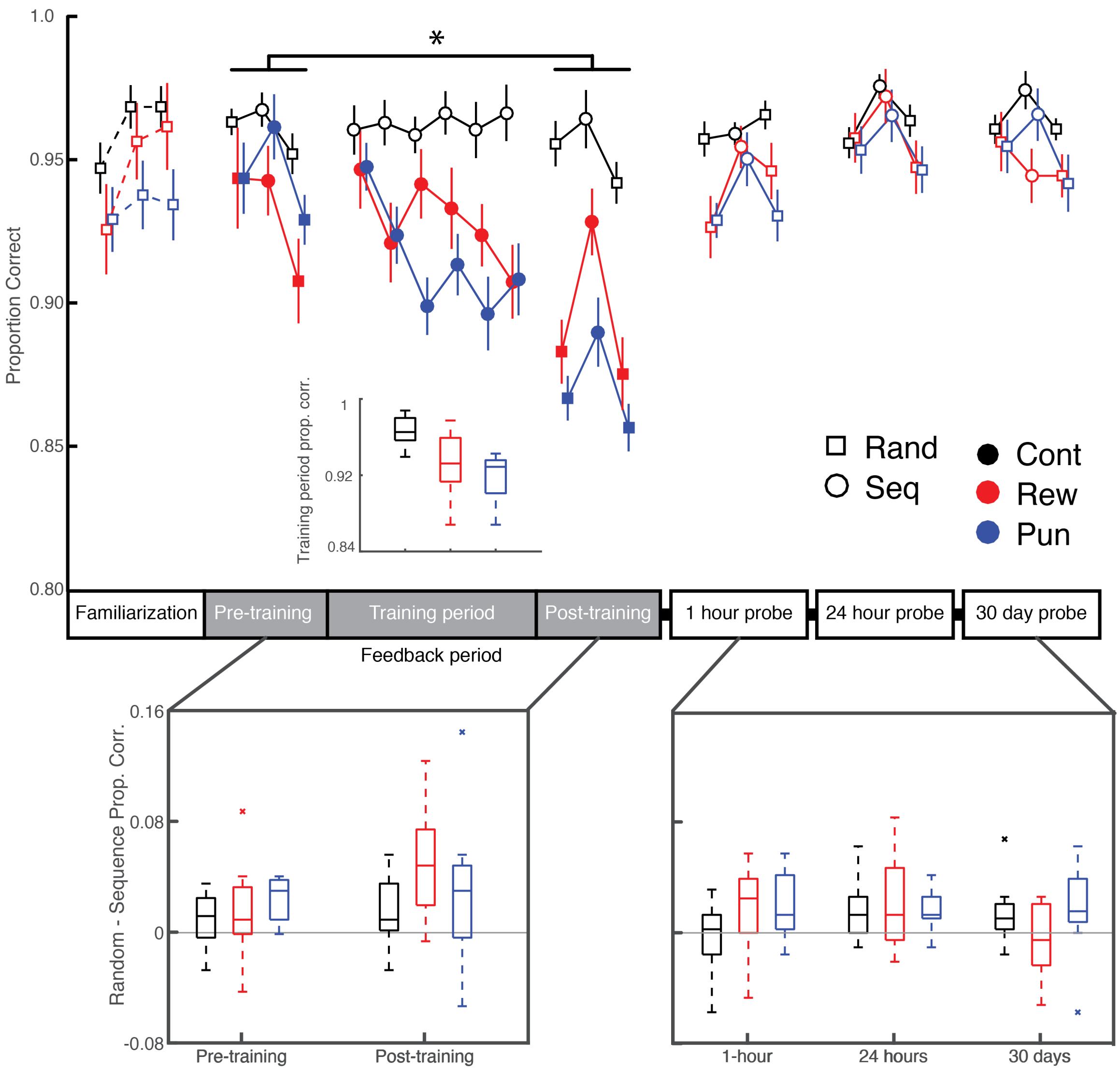

Figure S3. Error rate on the SRTT. Although the primary measure of behavior in the SRTT, for completeness we examined the accuracy during training. Before feedback was introduced, there was no significant difference between the feedback conditions ($F_{(2,35)}$ = 0.795, p = n.s.). We first compared the impact of feedback on the sequence knowledge after

learning by comparing the difference in the proportion of correct trials during the pre- and post- training probes using a repeated measures ANOVA, with Group (reward, punishment, control), Sequence (fixed, random), and Time point (pre, post) as factors. As expected, participants made less errors in fixed compared to random blocks (Main effect Sequence: $F_{(1,33)} = 40.474$, $p < 0.001$). Participants were less accurate overall during the post-training Time point (Main effect of Time point: $F_{(1,33)} = 37.832$, $p < 0.001$).

The type of feedback given during the probe blocks did effect the accuracy during these blocks (Main effect of Group: $F_{(1,33)} = 5.335$, $p < 0.01$). Post-hoc tests revealed that control was significantly more accurate than reward ($t_{(22)} = 2.743$, $p < 0.012$) and punishment ($t_{(22)} = 3.5$, $p < 0.005$), while reward and punishment did not differ ($t_{(22)} = 0.362$, $p = 3.720$.). Feedback condition showed a significant interaction with run ($F_{(2,33)} = 11.894$, $p < 0.001$). Post-hoc analyses revealed that reward and punishment made more errors during the post-training probe compared to the pre training probe, indicating that the sacrificed accuracy more than control (Reward v Control: $t_{(22)} = 2.658$, $p < 0.014$, $t_{(22)} = 4.562$, $p < 0.001$). The change in proportion correct from pre- to post- training was not significantly different between punishment and reward when correcting for multiple comparisons ($t_{(22)} = 2.547$, $p = 0.018$). There was no significant interaction between Group and Sequence ($F_{(2,33)} = 2.548$, $p = 0.093$) or for Group x Sequence x Time point ($F_{(2,33)} = 1.659$ $p = 0.206$)

As in reaction time, we examined the effect of feedback on the learning period by comparing accuracy across training period using a repeated measures ANOVA with Block and Group as factors. Participants tended to get less accurate over time (Main effect Block: $F_{(5,165)} = 5.078$, $p < 0.001$; block 1 v block 6: $t_{(35)} = 3.209$, $p < 0.005$). There was a main effect of feedback Group on accuracy during these blocks ($F_{(2,33)} =10.768$, $p < 0.001$). Post-hoc analysis revealed that punishment ($t_{(22)} = 5.462$, $p < 0.001$) and reward ($t_{(22)} = 3.140$, $p <$

0.005) were less accurate than control during the learning period. Reward and punishment did not differ in their error rates ($t_{(22)} = 1.182$, $p = 0.251$). There was also a significant Block by Group interaction ($F_{(10,33)} = 3.419$, $p < 0.001$). Follow-up analysis showed that punishment and reward showed a greater decrease in accuracy from block 1 to block 6 compared to control (Reward v Control: $t_{(22)} = 2.852$, $p < 0.01$; Punishment v Control: $t_{(22)} = 2.851$, $p < 0.05$) but were not significantly different from each other (Reward v Punishment: $t_{(22)} = 0.0$, $p = 1$).

Finally, to test whether the feedback Groups differed during the Delayed test probes, we compared the average accuracy during the fixed sequence test probes the accuracy during random sequence test probes using a repeated measures ANOVA with Probe, Group, and Sequence as factors. All subjects showed retention of sequence knowledge during the delayed probes, evidenced by elevated accuracy in fixed- compared to random- sequence blocks (Main effect of Sequence: $F_{(1,33)} = 24.492$, $p < 0.001$; $t_{(35)} = -4.954$, $p < 0.001$). There was also a main effect of Probe ($F_{(2,33)} = 6.814$, $p < 0.005$), indicating that subject's tended to be more accurate at the 30 day test compared to the 1-hour test ($t_{(35)} = -2.554$, $p < 0.05$). There was no effect of Feedback Group on accuracy in the delayed test probes.

S4. Conscious recall

We assessed conscious recall for the SRT, but not the FTT. However, because we did not reveal the presence of the sequence until 30 days after learning, we believed that verbal recall would be an insensitive measure to assess conscious knowledge in our task. Therefore, we implemented the process dissociation procedure in the same manner described by Song and Cohen [1]. These data are presented below. Briefly, after completion of the 30-day test probe, subjects asked to input 100 button-presses while trying to repeat as much of the sequence as they could recall. They were specifically instructed that if they thought they thought that they knew any of the sequence, or several chunks of the sequence, that they should input those pieces as many times as they felt that they could. The area under the curve for each number of sequential items (triplets, quadruplets, quintuplets through dodecs) included in the 100 button presses generated by the subject was compared to the chance, which was estimated via 10,000 Monte-Carlo simulations of 100 non-repeating items. For the simulations, item frequency was calibrated to each subject using the frequency of each button's appearance during 100 button presses where the subject was instructed to press buttons randomly with no repeats. The groups were compared using a one-way ANOVA with Group as a factor.

Using this method, we determined that there was no difference between the feedback groups ($F_{(2,35)} = 0.705$, $p = 0.501$). Data for all subject's area under the curve are plotted below. Prior work has found that subject's who are informed of the presence of a sequence prior to training have an average difference from chance of approximately 4 [1]. Thus, we believe that all subjects showed unconscious learning when assessed at 30 days after training.

Area under the curve difference between triplets generated during process dissociation procedure and chance level (mean ± SEM).

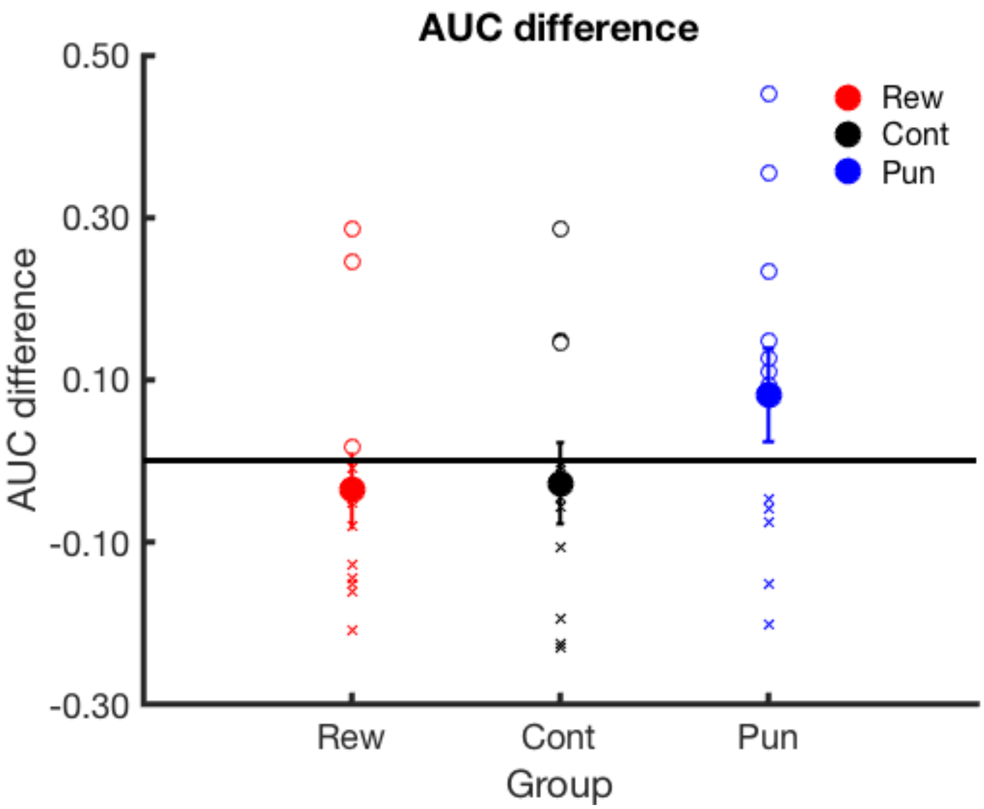

Trial-by-Trial performance on the FTT

We are happy to address this concern. To clarify, during the learning period, participants performed 8 repetitions of a single sequence during each of the 6 blocks. So each participant only sees one sequence during the training period.

For the SRT, we looked at the reaction time for each 12-item sequence repetition, excluding errors. For the SRT, these data are plotted below (mean ± SEM). Inset shows an example block of 8 sequence repetitions.

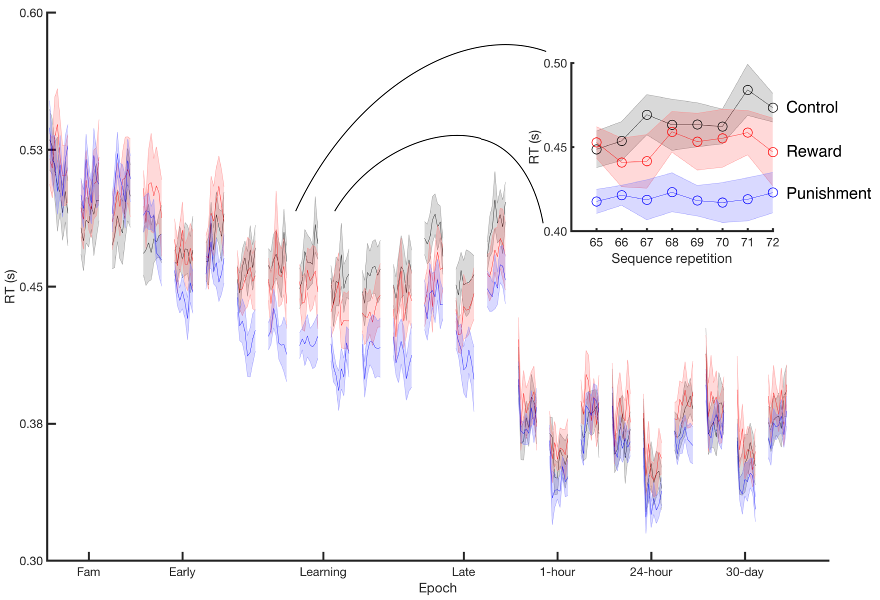

To explore the learning rate, for each participant we fit both linear and exponential functions to the reaction times for each sequence repetition during the learning time period (number sequence repetitions = 48). We compared the learning rate terms (slope for linear fit/exponent-term for exponential) of the participants in each group using a one-way ANOVA with Group (Control, Reward, Punishment) as a factor. There was no effect of group for either linear or exponential models [linear: ($F_{(2,35)}$ = 1.352, p = .273); exponential ($F_{(2.35)}$ = 1.129, p = 0.336)]. Neither the linear nor the exponential model were significant when the entirety of the training period (Familiarization, Early probe, Learning, and Late probe) was used [linear: ($F_{(2,35)}$ = 0.499, p = .612); exponential ($F_{(2.35)}$ = .470, p = 0.629)].

We next looked at the performance at each trial repetition during the learning period. For the FTT, these data are plotted below (mean ± SEM). Inset shows an example 8-trial block.

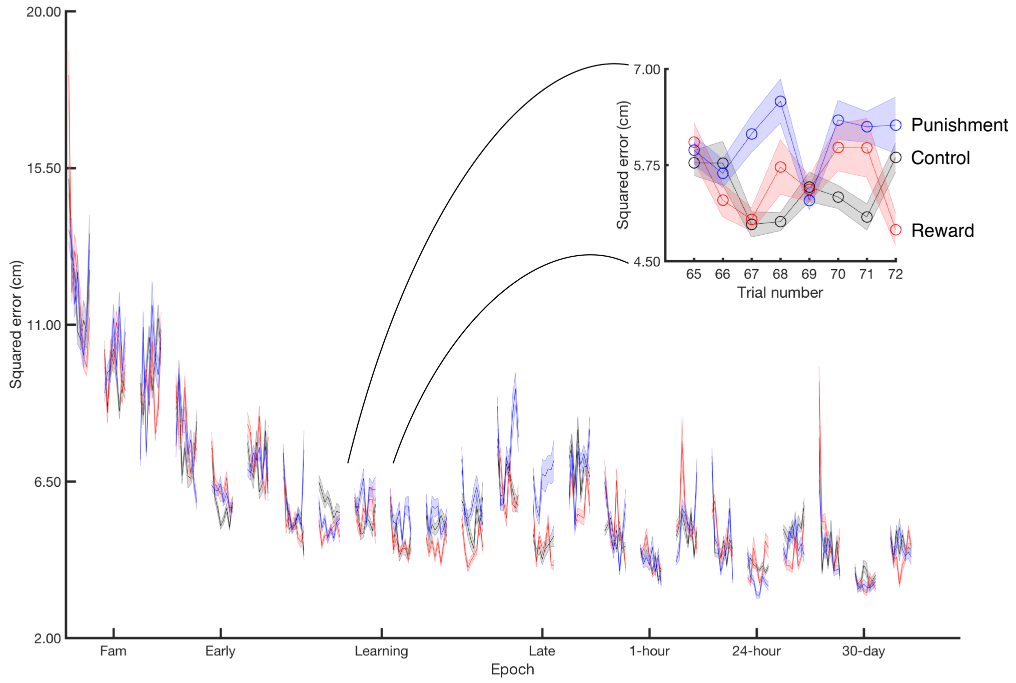

As performed for the SRT, for each participant we fit linear and exponential functions to the learning period (number of sequence trials = 48). We then compared the learning rate terms (slope for linear fit/exponent for exponential) for each group using a one-way ANOVA with Group (Control, Reward, Punishment) as a factor. There was no effect of Group in either the linear ($F_{(2,35)}$ = 1.274, p = 0.293) or exponential ($F_{(2,35)}$ = 1.201, p = 0.314) learning terms. This comparison was not significant when the entire training period (Familiarization, Early probe, Learning, and Late probe) were used to fit linear ($F_{(2,35)}$ = 1.420, p = 0.256) or the exponential models ($F_{(2,35)}$ = 1.413, p = 0.258).

References

1 Song, S. & Cohen, L. G. Conscious recall of different aspects of skill memory. *Frontiers in behavioral neuroscience* **8**, 233, doi:10.3389/fnbeh.2014.00233 (2014).
2 Hasson, C. J., Manczurowsky, J. & Yen, S. C. A reinforcement learning approach to gait training improves retention. *Front Hum Neurosci* **9**, 459, doi:10.3389/fnhum.2015.00459 (2015).
